# 

**Published:** 1871-02

**Authors:** 


ORIGINAL CONTRIBUTIONS.

Prostitution and its Sanitary Management. By Edmund Andrews, M.D. .... 65
Cancer of Testicle. By T. G. Williams, M.D., Watertown, Wis ........ 98
Counter-Irritation. By J. E, Hendricks, M.D.. DesMoines, Iowa, ........ 99
Pushing the Fœtus through the Pelvis. By S. B. Hawley, M.D., Aurora, Ill... 101
Paracentesis Tympani. By W. H. Fitch, M.D., Rockford, Ill. ........ 104

THE CLINIC.

Mercy Hospital Clinical Reports. By Prof. Davis. Case I. Typhoid Fever—Strychnia, Etc. .. ........ 107
Case II. Rheumatic Inflammation of Spinal Nerves, ........ 109

SELECTIONS.

Remarks on Degenerative Disease, or Atrophy, of the Gastric Tubules. By Austin Flint, M.D. ........ 111
Variola ten days after Successful Vaccination, ........ 97
Belladonna in Asthma, ........ 103
A Mechanical Anæsthetic, ........ 117
Extreme Contagiousness of Scarlet Fever 117
Pneumonitis, ........ 118
Sin the supposed Cause of Disease, ...... 118
Cardiac Murmurs in Chorea, ........ 118
Smallpox, ........ 119
Treatment of Ganglions, ........ 119
Scarlet Fever, ........ 119
Refracture of Bones, ........ 120
Mortality in Paris, ........ 120
The Pulse, ........ 120
The Originator of Pathological Anatomy 120
Iodide of Lead Ointment, ........ 120
Ipecacuanha in Dysentery, ........ 121

BOOK NOTICES.

A Report on the Barracks and Hospitals of the United States Army. By John S. Billings, Assistant-Surgeon, U. S. A. 121
The American Journal of Obstetrics and Diseases of Women and Children, ..... 123
Satan in Society. By a Physician, ....... 123

EDITORIAL.

The Social Evil, ........ 124
Medical College Commencements, ...... 124
Hydrate of Chloral—Large Dose—Recovery, ........ 125
Large Brain, ........ 125
Mortality for the Month of December 1870 126
Rain Fall with Comparative Mortality, 1869 and 1870. ........ 127
Money Receipts, ........ 128

